# Can reduced predation offset negative effects of sea louse parasites on chum salmon?

**DOI:** 10.1098/rspb.2013.2913

**Published:** 2014-02-07

**Authors:** Stephanie J. Peacock, Brendan M. Connors, Martin Krkošek, James R. Irvine, Mark A. Lewis

**Affiliations:** 1 Department of Biological Sciences, University of Alberta, Edmonton, Alberta, CanadaT6G 2E9; 2 School of Resource and Environmental Management, Simon Fraser University, Burnaby, British Columbia, CanadaV5A 1S6; 3 ESSA Technologies Ltd, Vancouver, British Columbia, CanadaV6H 3H4; 4 Department of Zoology, University of Otago, Dunedin 9016, New Zealand; 5 Salmon Coast Field Station, Simoom Sound, British Columbia, CanadaV0P 1S0; 6 Department of Ecology and Evolutionary Biology, University of Toronto, Toronto, Ontario, CanadaM5S 3B2; 7 Fisheries and Oceans Canada, Pacific Biological Station, Nanaimo, British Columbia, CanadaV9T 6N7; 8 Department of Mathematical and Statistical Sciences, University of Alberta, Edmonton, Alberta, CanadaT6G 2G1

**Keywords:** parasite, predation, functional response, salmon, sea lice, model

## Abstract

The impact of parasites on hosts is invariably negative when considered in isolation, but may be complex and unexpected in nature. For example, if parasites make hosts less desirable to predators then gains from reduced predation may offset direct costs of being parasitized. We explore these ideas in the context of sea louse infestations on salmon. In Pacific Canada, sea lice can spread from farmed salmon to migrating juvenile wild salmon. Low numbers of sea lice can cause mortality of juvenile pink and chum salmon. For pink salmon, this has resulted in reduced productivity of river populations exposed to salmon farming. However, for chum salmon, we did not find an effect of sea louse infestations on productivity, despite high statistical power. Motivated by this unexpected result, we used a mathematical model to show how a parasite-induced shift in predation pressure from chum salmon to pink salmon could offset negative direct impacts of sea lice on chum salmon. This shift in predation is proposed to occur because predators show an innate preference for pink salmon prey. This preference may be more easily expressed when sea lice compromise juvenile salmon hosts, making them easier to catch. Our results indicate how the ecological context of host–parasite interactions may dampen, or even reverse, the expected impact of parasites on host populations.

## 1. Introduction

By definition, parasites harm their hosts [1]. The fitness of parasitized individuals can decrease through direct parasite-induced mortality, reduced fecundity (for example, via parasitic castration [2]) or reduced reproductive success (for example, via sexual selection [3]). The impact of parasites on host individuals is invariably negative when considered in isolation, but may be complex and unexpected in nature. Parasitism is interdependent with other ecological interactions that the host experiences, such as predation and competition [4]. For example, parasites may increase host susceptibility to predation [5,6] and, in turn, parasite populations may be regulated when infested hosts are preyed upon [7]. Feedbacks between parasitism and predation can be further complicated by nonlinear predator–prey dynamics and clumping of parasites among hosts.

The direct impact of parasites on host population dynamics may be weak relative to other drivers such as predation because parasite infestations are often sub-lethal [4]. Therefore, understanding the indirect effects of parasitism on processes such as predation may actually be more important than understanding the direct effects of parasites on isolated host individuals. If parasites act to reduce predation on hosts then the net effect of infestation may be negligible or even positive for the host if gains from reduced predation offset or exceed direct costs of parasitism. Here, we explore this idea in the context of parasitic sea louse infestations of juvenile pink and chum salmon (*Oncorhynchus* spp.) in Pacific Canada.

### (a) Study system

*Lepeophtheirus salmonis*, commonly known as sea lice or salmon lice, are marine copepods that infest salmon and trout. Sea lice feed on host epidermis, musculature and blood, causing damage to host surface tissues that can lead to osmoregulatory stress [8], expose hosts to secondary infections [9] and cause host behavioural changes [6] or death [10,11]. Sea louse infestations may also have ecological impacts on wild salmon, particularly juveniles, as infested individuals have compromised schooling [6] and swimming abilities [12,13] and may be unable to complete migrations or evade predators [6]. Juvenile salmon experience very high predation rates during early marine life [14,15], suggesting that the effects of parasitism on predator–prey interactions may be important for evaluating the consequences of sea louse infestations on salmon population dynamics.

Pacific salmon are anadromous and semelparous species; they hatch in freshwater, migrate to sea to spend the majority of their lives and then return to freshwater to spawn and die. Outwardly migrating juvenile salmon are relatively free of sea lice, which cannot survive in freshwater. Juvenile salmon are not exposed to substantial numbers of sea lice until several months into their migration when they encounter returning adult salmon [16]. However in recent decades, salmon farms have provided a host reservoir population for sea lice that persists year-round in close proximity to salmon-bearing rivers. The high density of hosts on salmon farms can amplify natural infestations and sea lice can spill back from farmed salmon to infest juvenile wild salmon very early in the juvenile salmon migration [17]. Epizootics of sea lice on farmed salmon have been implicated in the decline of wild salmon in Pacific Canada [18] and Europe [19–22]. The expansion of salmon aquaculture [23] has therefore brought conservation concerns in regions where the narrow inlets occupied by salmon farms are important migratory corridors for wild salmon.

One such region is the Broughton Archipelago of British Columbia, Canada (see the electronic supplementary material, figure S1). The productivity of Broughton pink salmon (*Oncorhynchus gorbuscha*) declined concurrent with sea louse infestations on salmon farms in the early 2000s [18]. There remains uncertainty about the magnitude of these effects owing to the potential for unidentified confounding factors affecting salmon survival as well as both process and observation error. Conflicting reports (e.g. [18,24]) highlight the sensitivity of these results to model assumptions and error. Despite these uncertainties, or perhaps because of them, the evidence linking sea louse infestations to declines in pink salmon has triggered a flurry of scientific activity. Although research on the impacts of sea lice has burgeoned in the past decade, the effect of infestations on chum salmon (*Oncorhynchus keta*) productivity has not previously been reported.

Pink and chum salmon are often observed in mixed-species schools in the near shore marine environment only days after emergence from the gravel [25]. At this stage, pink and chum salmon are small in size (approx. 30 mm body length and 0.2 g weight) and have underdeveloped epidermal, immune and osmoregulatory systems [26]. Furthermore, they do not develop scales until several weeks after marine entry and therefore lack the mechanical resistance to sea louse attachment and feeding that scales may confer [27]. Because of their similar early life histories and comparable rates of direct parasite-induced mortality [11], both juvenile pink and chum salmon may succumb to even low levels of parasitism [17]. Therefore, we expected that chum salmon might show decreased productivity during a regime of sea louse infestations in the Broughton Archipelago, as was found for pink salmon [18].

In this study, we first report on an analysis of chum salmon spawner-recruit data in which we did not find an effect of sea lice on chum salmon productivity despite high statistical power. This unexpected result led us to investigate the effects of parasitism on interactions within a juvenile salmon food web that may mitigate the impact of sea lice for chum salmon. Predation by coho salmon (*Oncorhynchus kisutch*) is an important source of mortality for both juvenile pink and juvenile chum salmon [25] that may be mediated by sea lice [6]. Field-based experiments suggest that coho salmon prefer to consume pink salmon over chum salmon [28]. In the second part of the paper, we use a mathematical model to explore the conditions under which a parasite-induced shift in predation to pink salmon may lead to higher chum salmon survival in a regime of sea louse infestations. The results indicate that the ecological context of host–parasite interactions may alter, or even reverse, the expected impact of parasites on host populations.

## 2. Chum salmon productivity

### (a) Methods

In fisheries, productivity can be calculated as the mean number of offspring that survive to adulthood and are either caught in fisheries or return to freshwater to spawn (i.e. recruits per spawner) [29]. We modelled chum salmon productivity using a Ricker spawner-recruit model [29,30]. The full model included hierarchical terms to account for spatial and temporal covariation among populations (described below) and a covariate describing the effect of sea lice on chum salmon productivity [18]:2.1
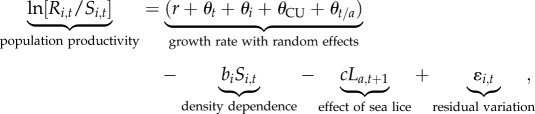
where *R_i,t_* are the recruits to population *i* produced by spawners in brood year *t*, *S_i,t_* is the spawner abundance, *r* is the overall growth rate, *b_i_S_i,t_* is the population-specific within brood year density-dependent mortality and *cL_a,t_*_+__1_ is the estimated mortality of chum salmon due to sea lice. Residual variation, *ɛ_i,t_*, is normally distributed with mean zero and variance to be estimated. We ignored measurement error associated with the enumeration of spawners, as in previous studies of spawner-recruit data in relation to sea lice (e.g. [18,31]), because accounting for both process and measurement error greatly complicates the analysis. Furthermore, it has been shown that explicitly including measurement error in a state-space framework does not improve parameter estimates in the range of growth rates that we encountered [32].

For species that return at different ages, such as chum salmon, recruits in a given return year need to be assigned to brood years based on the distribution of ages at return. Spawner-recruit time series by brood year for river populations on the south-central coast of British Columbia, Canada (see the electronic supplementary material, figure S1) were compiled from spawner, catch and age-at-return data provided by Fisheries and Oceans Canada [33]. River populations were excluded from the analysis if there were spawner abundance estimates for less than one-third of the years analysed. Chum salmon return to spawn as 3-, 4- or 5-year-old adults. The distribution of ages for multiple return years is therefore needed to calculate total recruitment corresponding to a single brood year. In the case of age-at-return data, we imputed missing values and investigated the sensitivity of our results to the assumptions of the imputation method. Details regarding the treatment of spawner-recruit data are provided in the electronic supplementary material. We analysed trends in productivity from 1980 to 2005 for 63 chum salmon river populations; 53 unexposed and 10 exposed to salmon farms (see the electronic supplementary material, table S1).

Adult Pacific salmon tend to return to their natal rivers to spawn [34], allowing us to analyse factors affecting chum salmon productivity at the spatial scale of river populations. However, chum salmon display lower fidelity to natal rivers than some other salmon species (e.g. sockeye salmon). We accounted for synchrony in productivity among river populations at larger spatial scales by modelling variability in growth rates among years, regions, statistical management areas and ecologically and/or genetically distinct biological units termed Conservation Units under Canada's Wild Salmon Policy [35,36]. Variability in growth rates among years common to all populations in the region was included as *θ_t_* [37,38]. Variability in growth rates among populations was included as *θ_i_* [39]. Variability in growth rates among Conservation Units was included as *θ*_CU_. Finally, variability in growth rates among Pacific fishery management areas was included as *θ_t/a_* and accounts for the non-independence of recruitment estimates within management areas owing to common harvest rates and the non-independence of sea louse abundance which is measured at the scale of management areas (see the electronic supplementary material, figure S1). These random effects were assumed to be normally distributed random variables with means of zero and variances to be estimated.

The covariate *L_a_*_,*t*__+__1_ is an estimate of parasite exposure for populations in area *a* and year *t* + 1 when juvenile chum salmon from brood year *t* enter the ocean and migrate past salmon farms. We investigated two different forms of this covariate (see the electronic supplementary material, table S2). First, we summed the number of adult female sea lice in April on all farmed salmon in the vicinity of the juvenile salmon migration route (see the electronic supplementary material, figure S1). For years 2000–2002, some of these salmon farms did not report sea louse abundances, and so we estimated these abundances under four different scenarios (F_1_–F_4_ [18]). The second form of the covariate was the average number of attached sea lice (copepodid, chalimus and motile stages) per juvenile wild pink and chum salmon [31]. Owing to the absence of data for sea louse abundances on farmed and wild salmon in the 1990s [24,31], brood years 1990–1998 for the farm sea louse covariates and 1990–1999 for the wild sea louse covariate were excluded from the analysis. We tested the significance of the sea louse covariate using a likelihood ratio test with the null model *c* = 0 indicating no correlation between sea louse abundance and chum salmon productivity. We performed a retrospective power analysis to determine our power to detect an effect of sea lice if an effect indeed existed. Details on how we calculated the sea louse covariates and the power analysis are provided in the electronic supplementary material.

### (b) Results

**Figure 1.**
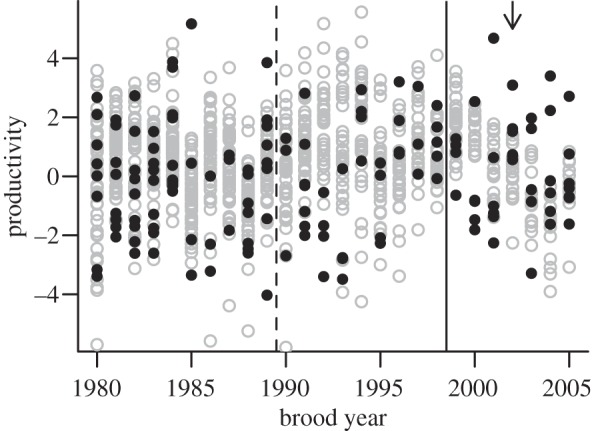
Productivity (log recruits per spawner) of chum salmon river populations in south-central British Columbia that were unexposed (grey open circles) or exposed to sea lice from farmed salmon in the Broughton Archipelago (black closed circles). Salmon farming was expanding in the Broughton Archipelago *ca* 1990 (dashed line), while the onset of recorded sea louse infestations was not until 2000 (affecting chum salmon from brood year 1999, solid line). A fallow management intervention in 2003 affected those salmon migrating from brood year 2002 (arrow).

There was no evidence of reduced productivity of chum salmon populations exposed to sea louse infestations on farmed salmon (see the electronic supplementary material, figures S2 and S3). The model fit was not improved by including a sea louse covariate (table 1). Populations exposed to salmon farms showed no obvious declines in productivity associated with either the expansion of salmon farming *ca* 1990 or sea louse infestations (1999–2005; figure 1). These results were consistent across all age-at-return scenarios that we considered (see the electronic supplementary material, table S4). We found significant covariation among populations within our study region in each year, within populations, Conservation Units and within management area each year, as indicated by an improvement of the model when all random effects were included (see the electronic supplementary material, figure S4).

**Table 1.** The parameter for sea louse-induced mortality of chum salmon, *c*, was not significantly different from zero for all forms of the sea louse covariate, *L_a_*_,*t*__+__1_ (equation (2.1)). (Likelihood ratio tests with the null model showed no improvement with the inclusions of the sea louse covariate. Results for different age-at-return scenarios are provided in the electronic supplementary material, table S4.)

louse covariate	*c*^a^	log-likelihoods^b^	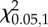	*p*-value
F_1_	0.064 (−0.025, 0.155)	−1615.1	3.601	0.058
F_2_	0.077 (−0.077, 0.222)	−1616.0	1.798	0.180
F_3_	0.068 (−0.054, 0.185)	−1615.8	2.260	0.133
F_4_	0.069 (−0.055, 0.189)	−1615.8	2.262	0.133
W	0.109 (−0.048, 0.251)	−1609.0	3.084	0.079

^a^Maximum-likelihood parameter estimate (95% bootstrapped CI).

^b^Log-likelihoods are not directly comparable among covariate models, as the wild and sea louse datasets had different amounts of missing data (see the electronic supplementary material, table S2).

The lack of a significant correlation alone is not reason to discount a possible impact of sea louse infestations on chum salmon populations. However, we had high power to detect changes in growth rate that would have resulted in population declines. Simulations incorporating model estimates of variability (see the electronic supplementary material) indicated we had 80.1–99.8% power to detect a rate of decline of *c* = 0.20, depending on the form of the covariate used (see the electronic supplementary material, figure S5). We had more than 70% power to detect effects in the range of those found for pink salmon [18,31], except for the F_1_ covariate, for which we had just 46.2% power to detect the effect size found for pink salmon. Although sea lice increase mortality rates of individual chum salmon in captivity [10,17], our results suggest that this does not translate to a measurable impact on chum salmon at the population level.

## 3. Parasite-mediated changes to predation

**Figure 2.**
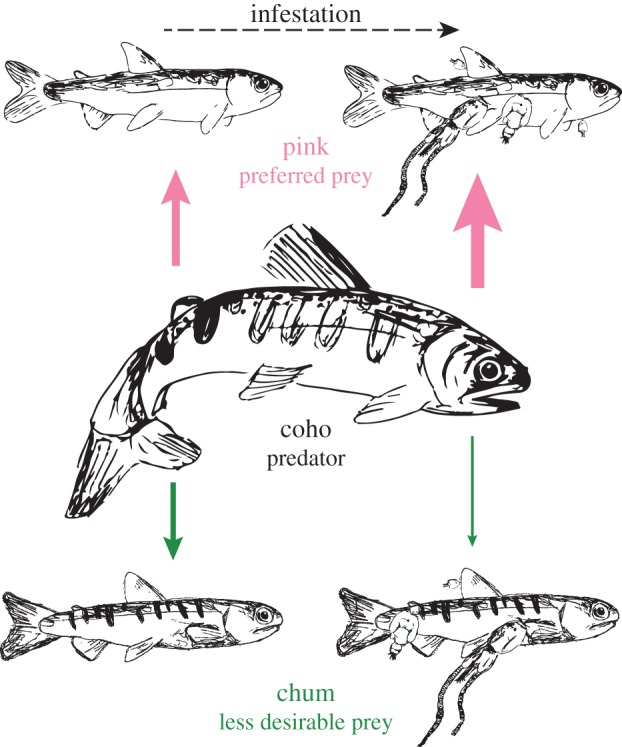
Predation pressure, indicated by the thickness of the solid arrows, is higher for pink salmon as they are a preferred prey of coho salmon [28]. As prey become parasitized, they are easier to identify and/or catch allowing predators to more easily express their prey preference. As a result, infestation may decrease predation pressure on less desirable prey species. In this case, chum salmon may experience lower predation pressure in a regime of sea louse infestations. (Online version in colour.)

In the following section, we develop a host–macroparasite model describing the population dynamics of a generalist parasite and two hosts in the presence of a common predator. Our objective is to determine the biologically relevant conditions under which reduced predation may lead to a negligible net impact of parasites on the survival of one of the host populations. The model has general applicability, but we employ parameters from the literature for sea lice and Pacific salmon hosts to determine whether parasite-mediated changes to predation may offset direct parasite-induced mortality for chum salmon (figure 2).

### (a) The functional response

The functional response describes the consumption rate of a predator as a function of prey abundance or density. Holling's type II functional response predicts increasing predation rates with increasing prey abundance to a satiation point, above which predators are limited by the time it takes to handle and digest prey [40]. For a predator of more than one prey species, consumption rates of a particular prey species are lower because the predator spends time handling alternative prey [41,42].

The influence of parasites on predator–prey interactions varies, depending largely on whether parasites make prey more or less vulnerable to predation [43]. Infested individuals may incur additional costs that require them to display riskier foraging behaviour or they may be physiological impaired and more likely to succumb to predation (e.g. [43,44]). We adapted the type II functional response to include a linear increase in the capture rate with increasing number of parasites:3.1
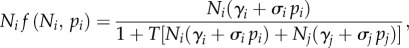
where subscripts *i* and *j* indicate parameters or variables associated with the focal and alternate prey species, respectively, *N* is the prey abundance, *γ* is the capture rate (i.e. rate of successful search resulting in consumption of the prey item), *T* is the handling time taken to consume and digest a single prey item, *σ* is the per-parasite increase in capture rate and *p* is the number of parasites per host [6]. We assume that the capture rate increases linearly with the number of parasites because for juvenile salmon, because of their small size, a single sea louse may have detrimental affects on performance. However, there may be thresholds in the number of parasites below which parasites have little effect on host behaviour, particularly for larger fish [45], and so we also consider nonlinear increases in capture rates over the number of parasites in the electronic supplementary material.

### (b) Host–parasite population dynamics

To evaluate the combined effects of parasite-mediated changes to predation and direct parasite-induced mortality, we consider the above functional response in a mathematical model describing the change in abundance of two host populations and each of their associated parasite populations. We treat predator abundance as a constant [43], independent of host/prey abundance and parasites. That is, we consider the functional response of predators to the abundance of prey but do not include a numerical response in the predator. This was in part to simplify the model, but also because numerical responses of coho salmon probably occur on much longer time-scales than the within-season dynamics of juvenile salmon hosts and parasites that we consider. The model builds upon the original host–macroparasite model by Anderson & May [1] and more recent work by Krkošek *et al*. [6] who considered just a single host species and associated parasite population. We present the basic equations of the model in the following section, but reserve mathematical details for the electronic supplementary material.

The first pair of equations describes the decline in abundance of two host populations, *N*_1_ and *N*_2_, owing to predation and direct parasite-induced mortality, where the host species interact through a common predator. The general equation for the change in population *i* in the presence of alternate prey *j* is3.2
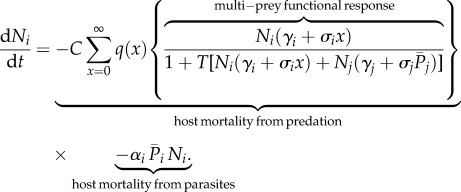


Host populations decline owing to predation at a rate predicted by the multi-prey functional response (3.1) and owing to direct parasite-induced mortality at rate 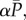 where 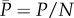 is the average number of parasites per host. As for the capture rate in (3.1), we assume that parasite-induced host mortality is linear over increasing parasite abundance, but we explore nonlinear thresholds in parasite-induced host mortality and per-parasite increases in capture rates in the electronic supplementary material. Host mortality depends on the number of parasites per host, therefore the average mortality rate of hosts is the sum of mortalities for all possible numbers of parasites from *x* = 0 to infinity, multiplied by the probability of a host having *x* number of parasites, *q*(*x*) [1]. There is no source term for *N_i_* because we considered the survival of a cohort of hosts, not including host reproduction. This approach is applicable to migrating juvenile salmon, and also avoids having to account for the potentially large difference in generation times of hosts and parasites.

The second pair of equations describes the change in the total number of parasites on each host population, *P*_1_ and *P*_2_. Once again, we present the general form for the total number of parasites on host/prey population *i*:

3.3
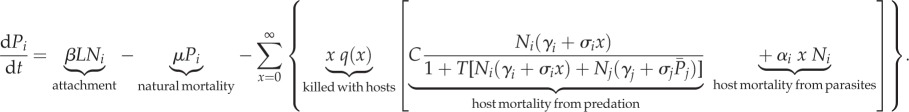


Parasites attach at rate *βL*, where *β* is the transmission coefficient and *L* is the density of free-living infectious-stage parasites. Parasites have a natural mortality rate, *μ*. We assumed that attachment and mortality were the same on both hosts populations, although this assumption could be relaxed for other systems. Finally, parasites were assumed to die with their hosts. Although it has been shown that parasites can be trophically transmitted from prey to predator [46], we do not consider the infection level of the predators in our model and so parasites that might jump onto successful predators are considered removed from the system.

The host–parasite model described by equations (3.2) and (3.3) could be applied to any pair of host species that share a common parasite and a common predator. We developed the model to investigate whether parasite-mediated changes to predation could offset direct effects of sea lice on chum salmon. When possible, parameter values for salmon and sea lice were drawn from the literature (table 2). For lesser-known parameters, we investigated the sensitivity of model output to a biologically reasonable range of parameter values. Details of our parameter selection are given in the electronic supplemental material.

**Table 2.** Parameters, symbols, and units used in the functional response and host–parasite population dynamics (equations (3.2) and (3.3)).

description	symbol	base value and units^a^	source
abundance of coho salmon predators	*C*	5000 predator	[6]
handling time	*T*	1 day	[6]
capture rate	*γ*_p_	3.40 × 10^–6^ (predator · day)^–1^	[6]
	*γ*_c_	2.72 × 10^–6^ (predator · day)^–1^	[6,28]
per-parasite increase in capture rate	*σ*_p_	5 × 10^–4^ hosts · (predator · parasite · day)^–1^	[6]
	*σ*_c_	5 × 10^–5^ hosts · (predator · parasite · day)^–1^	^b^
rate of parasite-induced host mortality	*α*	0.02 hosts · (parasite · day)^–1^	[11,17]
infection pressure	*βL*	0.05 parasites · (host · day)^–1^	[17]^b^
natural mortality rate of sea lice	*μ*	0.24 day^–1^	[11]
dispersion parameter	*K*	1.199	

^a^Basic physical dimensions of units are time for days and number of individuals for all other variables.

^b^Model output investigated under a range of values.

The survival of juvenile pink and chum salmon was calculated by numerically solving equations (3.2) and (3.3) using R [47] and the package *deSolve* [48]. The summation in equations (3.2) and (3.3) was numerically approximated for *x* = 0 : 1000. We assumed an initial population of *N*_1_(0) = *N*_2_(0) = 10^5^ salmon that leave a river with 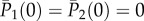 sea lice per host. The dynamics were simulated over the first 90 days of the juvenile salmon migration. This brief window was chosen because pink and chum salmon are the predominant prey of coho salmon starting when coho salmon follow the pink and chum salmon migration out of rivers into the near shore marine environment and ending six to eight weeks later when the prey outgrow their predator [25]. During this time, pink and chum salmon are also most susceptible to the effects of sea louse infestation [8]. Mathematical details and R code reproducing the simulations are available in the electronic supplementary material (see Data Accessibility).

### (c) Results

**Figure 3.**
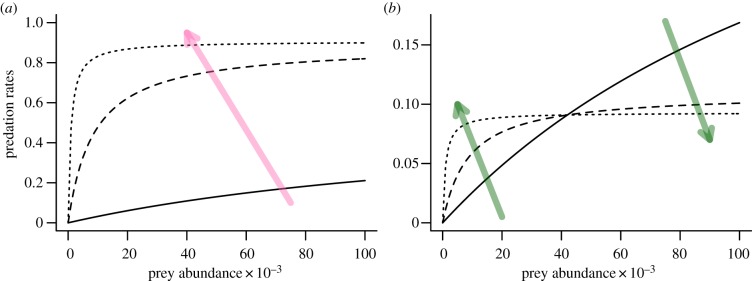
The proposed type II functional response of predation rates (prey consumed per predator per day; *y*-axis) on (*a*) pink salmon and (*b*) chum salmon over the number of prey available (*x*-axis) and parasite load (solid line 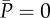, dashed line 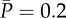, dotted line 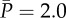). Light arrows indicate the direction of change in predation rates with increasing parasitism. For chum salmon (*b*), predation rates decrease with increasing number of parasites at higher prey abundance because predators can more easily capture their preferred prey—pink salmon. The abundance of alternate prey species was assumed to be the same as the focal prey species (*N_j_* = *N_i_*), and all other parameters were kept at their base values (table 2). (Online version in colour.)

The multi-prey type II functional response predicted higher predation rates on the preferred prey—pink salmon (figure 3*a*). Pink salmon made up proportionally more of the predators’ diet as parasite abundance increased because the per-parasite increase in capture rates of pink salmon was greater than for chum salmon. Overall predation rates on chum salmon, therefore, declined with increasing number of parasites when prey were abundant (figure 3*b*). However, at low prey abundance, predation rates were not in the saturation region of the type II functional response and parasites increased predation rates on both pink and chum salmon.

**Figure 4.**
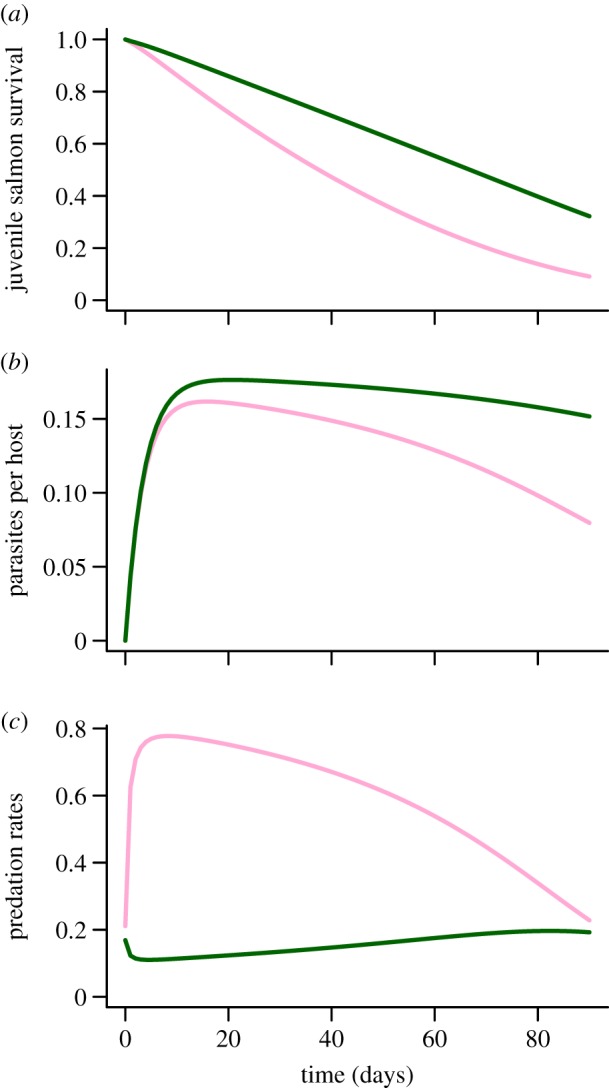
(*a*) Predicted survival of pink salmon (light line; pink online) and chum salmon (dark line; green online) over the period of juvenile salmon outmigration (*t* = 0–90 days). In our model, survival of pink and chum salmon declined with predation and direct parasite-induced mortality (equation (3.2)). (*b*) The predicted average number of sea lice per pink salmon (light line; pink online) and per chum salmon (dark line; green online), which changed with sea louse attachment, natural mortality and host mortality (equation (3.3)). (*c*) Predicted predation rates on pink salmon (light line; pink online) and on chum salmon (dark line; green online) over the period of the migration according to equation (3.1). Parameter values are given in table 2. (Online version in colour.)

These changes in predation rates with the number of parasites were reflected in the population dynamics of pink and chum salmon and associated sea lice. The survival of chum salmon was greater than the survival of pink salmon with base parameter values (figure 4*a*). The average number of sea lice was also greater on chum salmon than on pink salmon (figure 4*b*) because predators preferentially culled infected pink salmon. Predation rates on pink salmon increased steeply as the number of parasites per host increased at the start of the migration, whereas predation rates on chum salmon decreased initially (figure 4*c*). As parasitized individuals were removed from the population over the course of the migration and juvenile salmon abundance declined, predators were less able to focus on preferred prey and predation rates on pink salmon declined. Near the end of the migration, chum salmon were more abundant, had a higher parasite load and experienced similar predation rates as pink salmon.

The prediction of higher sea louse abundance on chum salmon (figure 4*b*) was supported by data from a long-term monitoring programme of sea lice on juvenile salmon in the Broughton Archipelago. We found higher average numbers of copepodid and chalimus sea lice on juvenile chum salmon than on pink salmon caught in the same sample (see the electronic supplementary material, table S5). Numbers of motile sea lice did not differ significantly between host species, but motiles are known to move among hosts in search of mates [49] or when their host is attacked by a predator [50].

**Figure 5.**
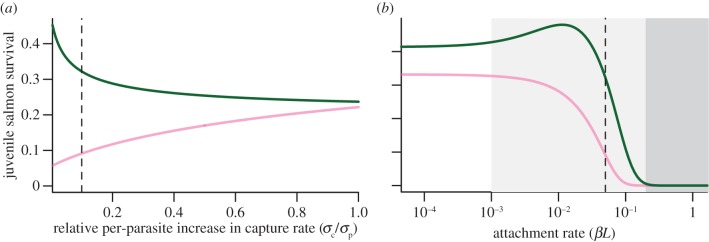
Predicted survival of pink salmon (light lines; pink online) and of chum salmon (dark lines; green online) as a function of (*a*) the ratio of per-parasite increase in capture rate for chum salmon over that for pink salmon, and (*b*) infestation pressure (i.e. attachment rate *βL*). Low, moderate and high infestation pressure are indicated by light, medium and dark grey shading in (*b*). Base parameter values are indicated by vertical dashed lines (table 2). (Online version in colour.)

In our model, the per-parasite increase in capture rates was relatively large compared with the base capture rates. Our results were therefore extremely sensitive to the relative per-parasite increase in capture rates for pink and chum salmon. If we assume the per-parasite increases in capture rates are the same for both pink and chum salmon, survival is similar for the two species even though pink salmon are far more likely to be captured in the absence of parasites (figure 5*a*). This is because the difference in overall capture rates between pink and chum salmon gets smaller as the number of parasites increases. However, if the per-parasite increase in capture rate is higher for pink salmon then for chum salmon, the difference in overall capture rates between pink and chum salmon grows as the number of parasites increases. Therefore, the assumption that predators such as coho salmon will focus predation on pink salmon as they become parasitized is essential for chum salmon survival to increase with infestations.

The population dynamics of juvenile salmon and sea lice were also sensitive to different levels of sea louse infestation pressure. Experimental work in the absence of predators indicates that survival of juvenile salmon declines with as few as one sea louse per fish [10,17]. We might expect that as infestation pressure increases, modelled by an increase in the attachment rate, *βL*, direct parasite-induced mortality may become important and survival of chum salmon would decline. We found that at low infestation pressure, survival of chum salmon was greater than the survival of pink salmon. At moderate infestation pressure and base parameter values (table 2), survival of pink salmon declined steeply as predation on pink salmon increased (see the electronic supplementary material, figures S6 and S7) but the survival of chum salmon actually increased because of reduced predation (figure 5*b*). This increase in chum survival was because of lower predation pressure at the beginning of the migration, when prey were still abundant and a reduction in predation rate had a large impact on the number of chum salmon consumed. As infestation pressure increased above the base value of *βL* = 0.05, survival of chum salmon declined to match that of pink salmon for two reasons: steeper declines in survival of pink salmon meant a low abundance of preferred prey, resulting in higher predation on chum salmon earlier in the migration (see the electronic supplementary material, figure S7) and direct parasite-induced mortality of chum salmon became increasingly important at high infestation pressure. This highlights the sensitivity of our results to infestation pressure, with the survival of chum salmon increasing only at moderate infestation intensity.

All of the results presented thus far refer to the model in which capture rates and host mortality increase linearly with the number of parasites. Some studies suggest that there may be thresholds for impact depending on the number of parasites [45] and the size of the hosts [8]. When the linear assumption was relaxed to include sigmoidal responses in capture rates and host mortality rates to the number of parasites (see the electronic supplementary material, figure S8), the results were largely unchanged; chum salmon survival and associated sea louse abundance were consistently higher than those for pink salmon (see the electronic supplementary material, figure S9). Additional results of nonlinear responses of hosts to parasites load are discussed in the electronic supplementary material.

## 4. Discussion

Parasites are generally considered a villainous guild, causing host morbidity and mortality. However, we hypothesized that in certain situations, the net effect of parasitism on hosts may be nullified or possibly positive when considering indirect effects of parasites on predator–prey interactions within a multi-host community. For communities of juvenile salmon, the physiological impact of sea louse parasitism has been well studied [8], and both pink and chum salmon in captivity show decreased survival with as few as one attached sea louse [10,11]. Multi-year studies of pink salmon population abundance data indicate that the net impact of sea louse infestations on pink salmon is probably negative [18], suggesting that direct parasite-induced mortality translates to reduced productivity of affected populations for pink salmon. However, our analysis of chum salmon population abundance data suggests the existence of an ecological mechanism that confers resilience to chum salmon populations despite the direct effects of infestations on host individuals. Indirect ecological effects of sea lice on salmon predator–prey interactions may be a key determinant of host survival. Sea louse parasites are known to increase the susceptibility of juvenile pink and chum salmon to predation [6] and coho predators prefer to consume pink salmon over chum salmon [28]. If infestations intensify predation on pink salmon, this may partially release chum salmon from predation, offsetting direct mortality costs of parasites on chum salmon.

Using a host–macroparasite model, we evaluated the conditions under which parasite-mediated changes to predation may offset direct impacts of parasites on host populations. We considered predation by a generalist predator (coho salmon) on two prey populations (pink and chum salmon) that share a common parasite (sea lice). Our model built upon previous experimental evidence that coho salmon predators exhibit a strong preference for pink salmon over chum salmon, even when pink salmon are larger (i.e. harder to catch) and less abundant than chum salmon [28]. The model allowed parasites to cause an increase in predation rates that was larger for the preferred prey, which can reduce predation on the other less desirable prey in the saturation region of the type II functional response. The less desirable prey had higher survival in regimes of moderate-intensity infestations. However, if the intensity of infestations was high enough, the negative direct impact of parasites overwhelmed any gains from reduced predation on the less desirable prey (figure 5*c*). Therefore, the potential indirect benefits that parasites may confer to hosts are probably constrained to a limited range of infestation levels.

Interestingly, the model predicted that the prey population with higher survival also had the higher parasite abundance. We understand this counterintuitive feature as follows: predation was focused on the preferred prey species and on parasitized individuals. This group of prey—preferred and parasitized—had the lowest survival. Less desirable prey species with parasites had higher survival and therefore the mean number of parasites per host was higher for less desirable prey (figure 3). In this case, the survival of hosts influenced their parasite load, rather than the parasite load influencing host survival, a reverse direction of causality than is usually assumed. This highlights the interdependence of parasite and host survival, and that host survival is not necessarily negatively related to the number of parasites.

The effects of sea lice on juvenile chum salmon survival are sensitive to the level of infestation pressure. An increase in chum salmon survival with sea lice only occurred over a moderate range of infestation levels. However, studies of infestation pressure from salmon farms in the Broughton Archipelago suggest that this may be the range of infestations that have occurred over the past decade [17,51]. At these moderate levels of infestation, pink salmon may experience significant mortality because parasites increase predation and thus mortality of pink salmon. Meanwhile, chum salmon populations may incur lower overall mortality because the redirection of predation mortality onto pink salmon caused by sea lice compensates or exceeds the direct impact of sea lice on chum salmon mortality. At high infestation levels, however, the model predicts a decline in chum salmon survival owing to overwhelming direct parasite-induced mortality. The sensitivity of both pink and chum salmon survival to high infestation levels in our model highlights the potential for decline in both pink and chum salmon populations should epizootics occur at sufficiently high levels. From a conservation perspective, it is therefore important to reduce abundances of sea lice in coastal regions shared by wild salmon and aquaculture.

The form of the functional response may influence the model outcome. We chose a type II functional response that has been used previously for salmon and sea lice [6] and has been recommended more generally for piscivorous fishes that actively pursue prey [52]. Coho salmon are active predators that prey on schooling pink and chum salmon. Nonetheless, different functional responses may alter the outcome of predator–prey interactions. For example, a type III functional response has decreasing capture rates when prey abundance is low because prey may be better able to seek refuge or the predator may shift focus to more abundant prey species [52]. This may result in much higher predation on chum salmon if they outnumber the alternate prey, even if the alternate prey are preferred.

Nonlinear changes in host capture rates and survival with sea lice are also worth consideration. Studies of the physiological impact of sea louse infestation on salmonid smolts indicate thresholds in louse abundance below which the impact is negligible [45]. However, for studies of juvenile pink and chum salmon, the presence of thresholds depends on the size of the host [8]. For salmon less than 0.5 g in weight, a single sea louse can reduce swimming performance [13], trigger measurable physiological changes [26] and cause mortality [10]. However, as juvenile salmon grow and develop scales, they can survive low levels of infestation with little effect. Our study focuses on juvenile salmon in the first two to three months of their migration when they are the primary prey for coho salmon predators. During this period, they are mostly below the 0.5 g threshold [8]. In the absence of more detailed studies of nonlinear effects of sea lice on such small hosts, we continued with the assumption of linear increases in host mortality and capture rates with the number of attached sea lice. We explored sigmoidal responses in the electronic supplementary material, but the main results were unchanged.

Coho salmon are major predators of juvenile pink and chum salmon [15,25], but also prey upon other species of fishes (e.g. Pacific herring, sand lance) and zooplankton [53]. We ignore these other prey species in our analysis and focus on predation rates on pink and chum salmon. Including additional prey species would not affect our results unless the alternative prey were both more numerous and preferred by coho salmon. Coho salmon often follow pink and chum salmon out of the rivers, and pink and chum salmon dominate the coastal ecosystem over the subsequent weeks [25]. Therefore, pink and chum salmon are probably the primary prey for coho salmon until they outgrow their predators six to eight weeks later [25].

There may be explanations for our inability to detect an effect of sea lice on the productivity of chum salmon other than parasite-mediated predation. First, chum salmon often return to larger geographical areas than specific rivers, potentially blurring the differences in survival between river populations exposed and not exposed to sea lice from farmed salmon. Chum salmon that emerge from a river outside the region of salmon farming will not pass by salmon farms as susceptible juveniles, but may return to a river within the region of salmon farming. High survival of such fish may confound a decline in survival of chum salmon migrating past salmon farms. Conservation Units, defined by Fisheries and Oceans Canada, include river populations with similar genetic and life-history traits, suggesting gene-flow among river populations within a Conservation Unit [36]. The Conservation Unit for chum salmon in the Broughton Archipelago includes river populations exposed and unexposed to salmon farming. However, the Conservation Unit for pink salmon in the Broughton Archipelago also includes both river populations exposed and unexposed to salmon farms [36], and yet the correlation between sea louse infestations on salmon farms and pink salmon survival in the Broughton Archipelago was significant [18]. However, if the effect size for chum salmon were smaller than for pink salmon, this movement of spawners may bolster survival of exposed river populations just enough to conceal any real impact of sea lice on chum salmon survival.

Second, inaccuracies in the chum salmon data may introduce uncertainty, making it harder to detect a statistically significant effect of sea lice. Fisheries and Oceans Canada aims to enumerate as many salmon species as possible while minimizing the cost of stock assessment programmes. Chum salmon may be counted at sub-optimal times, because they are usually the latest species to return within the season. Observation error is probably large because of the nature of enumeration methods (e.g. helicopter flights, stream walks). Return estimates do not include catch of chum salmon in First Nation fisheries or unreported catch of chum salmon in fisheries targeting other species [54]. Variable age-at-return in chum salmon introduces the potential for additional error that is not present in analyses of pink salmon population, which have a consistent 2-year life cycle. While our data on age-at-return for populations exposed to salmon farms were limited, our results were robust to different imputation methods for missing age-at-return. As a base case, we imputed missing age-at-return data with a constant 4-year age-at-return as this assumption minimizes spurious autocorrelation and cross-correlations between time series for different river populations [37]. Although these sensitivity analyses and a power analysis indicate our results are robust, we cannot ignore that errors accumulate with the different types of data we drew on in the spawner-recruit analysis.

The role that parasites are traditionally cast in is changing as we uncover the influence of parasites on competitive or predator–prey interactions in the host community [4]. Thinking beyond the direct impact of parasites on hosts is particularly important in the context of species conservation, where multi-host dynamics are often a necessary ingredient for disease to threaten biodiversity (i.e. reservoir hosts [55]). While results of theoretical models such as ours are sensitive to certain parameters and assumptions, they provide valuable insight into host–parasite dynamics under different ecological conditions. Our results here indicate that parasite effects on predator–prey interactions in multi-host dynamics may sometimes protect, or even enhance, the persistence of some host species, but this occurs at the expense of other species.

## Data accessibility

Chum salmon escapement, catch and age-at-return data and R code to reproduce analyses and model simulations can be downloaded at http://hdl.handle.net/10402/era.37172.
